# AI‐Driven Identification of Exceptionally Efficacious Polypharmacological Compounds That Extend the Lifespan of *Caenorhabditis elegans*

**DOI:** 10.1111/acel.70060

**Published:** 2025-04-22

**Authors:** Konstantin Avchaciov, Khalyd J. Clay, Kirill A. Denisov, Olga Burmistrova, Michael Petrascheck, Peter O. Fedichev

**Affiliations:** ^1^ Gero PTE Singapore Singapore; ^2^ Department of Molecular and Cellular Biology Molecular Medicine and Neuroscience, The Scripps Research Institute La Jolla California USA

**Keywords:** aging, artificial intelligence, Chemprop, drug discovery, geroprotector, GPCRs, longevity, machine learning, polypharmacology

## Abstract

Analysis of existing lifespan‐extending geroprotective compounds suggested that polypharmacological compounds are the most effective geroprotectors, specifically those that bind multiple biogenic amine receptors. To test this hypothesis, we used graph neural networks to predict polypharmacological geroprotectors and evaluated them in *Caenorhabditis elegans*. Over 70% of the selected compounds extended lifespan, with effect sizes in the top 5% compared to all geroprotectors recorded in the DrugAge database. Thus, our study reveals that rationally designing polypharmacological compounds enables the design of geroprotectors with exceptional efficacy.

## Graphical abstract

A retrospective analysis of the most effective known geroprotectors shows that they act by polypharmacological mechanisms. Using graph neural networks, we predict polypharmacological geroprotectors, validate their polypharmacological mechanisms experimentally, and show them to be exceptionally effective. Thus, it is possible to rationally design polypharmacological geroprotectors with exceptional efficacy.

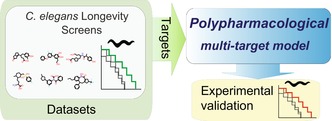

The genetic foundation of lifespan is becoming increasingly well‐understood, but the optimal strategies for designing interventions to extend it remain unclear. Small molecule drugs, the mainstay of the pharmaceutical industry, act by modulating the activity of gene products—proteins, herein referred to as targets. Standard drug‐discovery practice dictates that therapeutic compounds should be highly specific to a single target. However, closer inspection of FDA‐approved drugs reveals that some of the most efficacious drugs bind multiple targets simultaneously and that, in some instances, more specific analogs are less efficacious (Roth et al. 2004; Sexton and Christopoulos 2018). These findings suggest polypharmacology may improve efficacy for some complex indications.

The largest unbiased longevity screen of the Library of Pharmacologically Active Compounds (LOPAC), particularly FDA‐approved drugs, identified a significant cluster of compounds that extend lifespan by modulating neuroendocrine and neurotransmitter systems (Carretero et al. 2015; Ye et al. 2014). We observed that most inhibitors of G‐protein coupled receptors (GPCRs) bind multiple structurally related targets, suggesting that polypharmacological binding increases their efficacy in extending lifespan. To test this notion, we used statistical and machine learning tools, specifically graph neural networks (GNNs), to identify geroprotector compounds that simultaneously bind multiple biogenic amine receptors and then evaluated their efficacy on the lifespan of *Caenorhabditis elegans* (Figure 1A).

**FIGURE 1:**
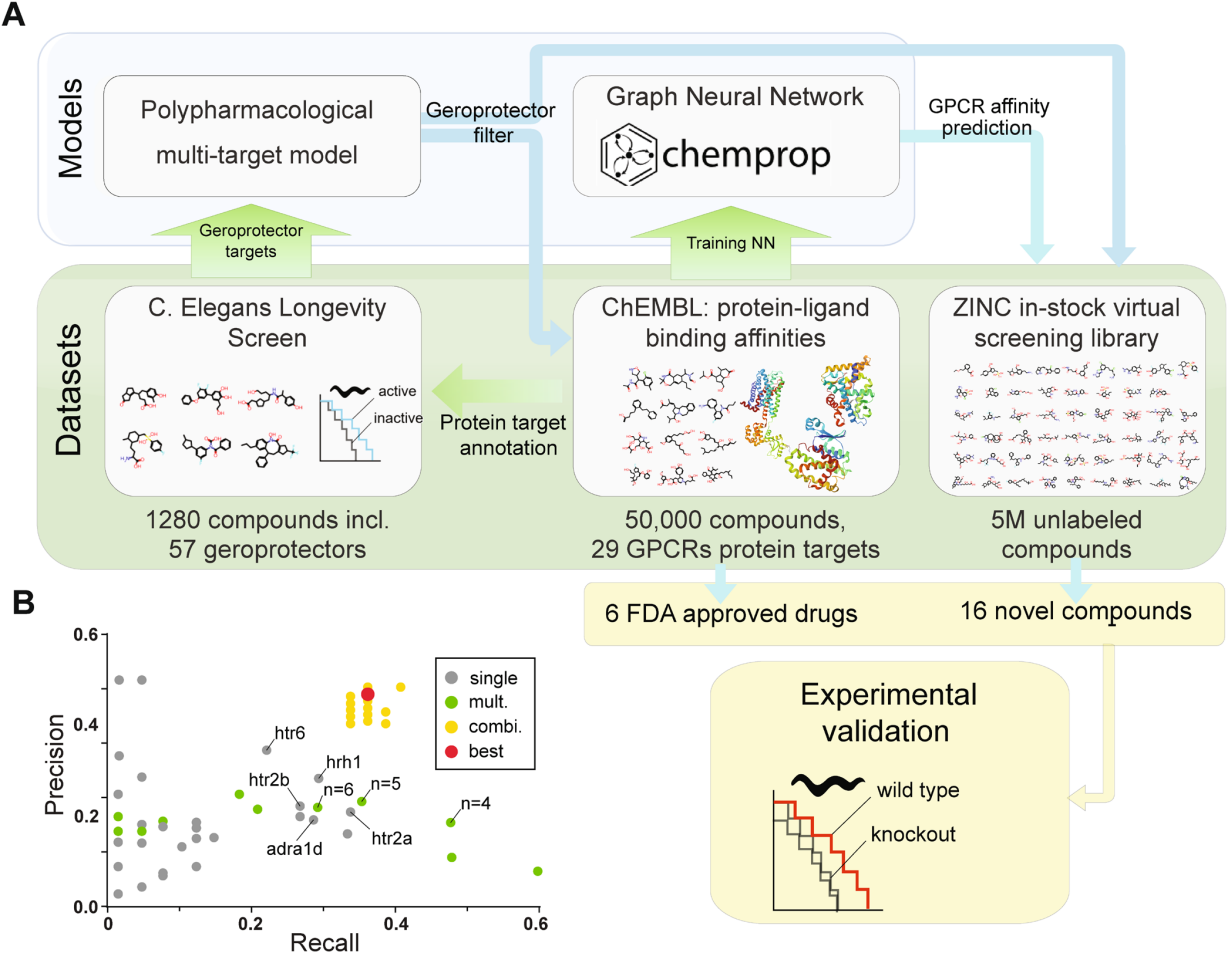
Computational design of lifespan‐extending polypharmacological geroprotectors. (A) Overview schematic of computational pipeline identifying polypharmacological compounds and validation in *Caenorhabditis elegans*. (B) Precision–recall scatter plot for the models implying either single cluster activity (gray dots), multiple clusters activity (green dots), and optimized combination of multiple clusters (yellow dots). Red indicates the optimized polypharmacological model selected for downstream analysis and experimental validation. Text labels indicate the leading target in the family of structurally related targets and the number of simultaneously identified binding activities for blue and green, respectively.

First, we annotated compounds from the LOPAC lifespan screen with target binding data from the ChEMBL database (Bento et al. 2014; Davies et al. 2015), a curated database of bioactive drug‐like molecules. Proteins were grouped in clusters based on sequence similarity, and we assumed that a compound annotated to bind to a single target within the cluster interacts with the whole cluster. Next, we evaluated the lifespan‐extending ability of the different clusters using a binary classification model, where the actual classification is the geroprotective, lifespan‐extending property of a compound (denoted LS+), and the predicted classification is the binding of a compound to any single protein target within a specified cluster.

We scored different aspects of model performance by standard precision/recall metrics and correlation (Matthews correlation coefficient [MCC]). Precision characterizes the model's accuracy by measuring the proportion of correctly identified true lifespan‐extending compounds. Recall, or sensitivity, calculates the proportion of accurately predicted positive outcomes from all actual positives, evaluating the model's ability to detect all true instances of lifespan extension. This evaluation identified 10 prominent clusters that, when targeted, affect lifespan significantly. We denoted these geroprotector‐target‐clusters (GT clusters). We named each cluster according to a key target within that cluster but used lowercase to distinguish the cluster (hrh1) from the target protein (HRH1) (Table S1).

The best correlation score (MCC = 0.24) between binding to a GT cluster and lifespan extension LS+ was observed for the htr6 cluster, which contains a single 5‐hydroxytryptamine receptor 6 (HTR6) gene. The best recall score was observed for the htr2a cluster, which includes the 5‐hydroxytryptamine receptor 2A/C (HTR2A, HTR2C) genes. Finally, the best combination of recall and precision score was observed for the hrh1 cluster, which contains a single histamine receptor H1 (HRH1) (Figure 1B, gray dots; Table S1).

We next asked whether polypharmacological compounds that bind combined sets of GT clusters would improve the predictive power of a model compared to binding a single cluster. We evaluated the predicted classification by varying the number of GT clusters a compound simultaneously binds to from 2 to 13 clusters. Figure 1B illustrates the recall–precision relationships for polypharmacological models binding multiple clusters (green dots, Table S2), compared to models representing highly selective compounds that bind to a single cluster (gray dots). We found that polypharmacological compounds binding four or five GT clusters increased the recall score but without a gain in precision scoring.

To further optimize recall–precision scores, we set out to identify the ideal combination of GT clusters to which a geroprotector compound should bind. We scored models in which a compound bound varying sets of GT clusters and generated predicted classifications for all possible combinations of a compound binding up to five GT clusters (yellow dots, Table S3). Polypharmacological compounds that simultaneously bound targets within the *drd2*, *hrh1*, and *htr6* clusters performed best (Figure 1B, red dot). Taken together, our analysis suggests that polypharmacological compounds binding the specific combination of *drd2*, *hrh1*, and *htr6* GT clusters would outperform single‐target compounds.

Next, we used this optimized polypharmacological model to computationally predict polypharmacological geroprotectors with activities across GPCR clusters using the ChEMBL compound database. A key problem in making polypharmacological predictions is that most ChEMBL compounds have limited polypharmacological profiling data available, as their pharmacological evaluation was restricted to one or only a few targets. To address the problem of sparse binding activity in the ChEMBL database, we leveraged modern machine‐learning algorithms to infer the binding of known drugs and novel uncharacterized compounds to GT clusters. Specifically, we used Chemprop, a GNN utilizing the Directed Message Passing Neural Network (D‐MPNN) architecture (Heid et al. 2024), to analyze 29 GPCR targets and 50,000 activity records to predict structures that bind to optimized combinations of GT clusters.

To experimentally validate the predictive capabilities of our polypharmacological model, we applied it to a curated list of experimental and approved compounds from ChEMBL, which bind at least two of the three *drd2*, *hrh1*, and *htr6* GT clusters simultaneously. This resulted in 296 unique compounds (Table S5), from which we selected six FDA‐approved drugs. Next, we used the trained Chemprop model to screen the ZINC20 library (Irwin et al. 2020) of purchasable compounds. After in silico filtering for drug‐likeness and removal of known geroprotectors, we selected a total of 22 novel structures to be tested for lifespan extension in *C. elegans* .

We found 16 compounds that significantly extended the lifespan of *C. elegans* at one or more doses (Figure 2A,B). The hit rate of over 70% (16/22) was approximately 1000‐fold higher than that of an unbiased screen and more than sevenfold higher than specifically targeting biogenic amines [29/333 in the LOPAC screen (Carretero et al. 2015)]. Thus, a polypharmacology approach significantly outperforms selection based on pharmacology class. Similarly, the effect sizes of the current series of compounds were significantly larger than any previous screens. Of the 16 hits, 12 extended lifespan by over 40% and eight by more than 50%. The mean lifespan extension of the 16 hits exceeded 48%, significantly more than the mean lifespan extension reported for all geroprotectors in DrugAge (~19%) (Barardo et al. 2017). Only 4% of geroprotectors annotated in DrugAge extend the lifespan of *C. elegans* by 44% or more, thus establishing our set in the top 5% of all known lifespan‐extending geroprotectors.

**FIGURE 2:**
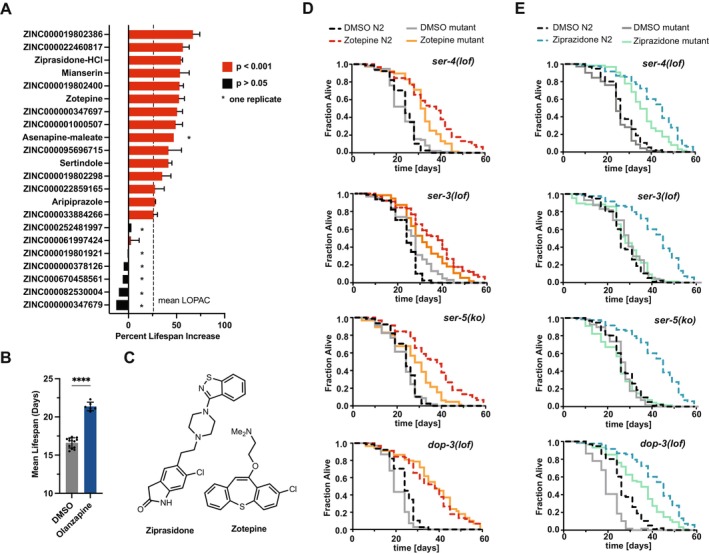
Computationally designed geroprotectors target multiple G‐protein coupled receptors. (A) Mean percent increase in lifespan extension observed at the optimal dose for two experiments for the 22 molecules designed. Mianserin was included as a blinded positive control. Error bars indicated mean ± SD. The mean lifespan extension observed for hits in the LOPAC screen is indicated by a dotted line to compare the effect sizes between a phenotypic screening strategy and our current strategy. The number of animals ranges from 35 to 93 per cohort. See Table S11 for statistics. (B) Olanzapine was used as an intra‐plate positive control that consistently extended lifespan across the screen. The unpaired, two‐tailed Student's *t*‐test determined significance where ****p < 0.0001. Error bars indicated mean ± SD. (C) Chemical structures of ziprasidone and zotepine. (D) Graphs show survival plots of *Caenorhabditis elegans* wild type (N2) or mutant animals harboring loss of function mutations in their respective receptor genes (*ser‐3*, *ser‐4*, *ser‐5*, and *dop‐3*), each treated with either DMSO or zotepine (10 μM). The number of animals ranges from 55 to 173 per cohort. See Table S12 for statistics. (E) Graphs show survival plots of *C. elegans* wild type (N2) or mutant animals harboring loss of function mutations in their respective receptor genes (*ser‐3*, *ser‐4*, *ser‐5*, and *dop‐3*), each treated with either DMSO or ziprasidone (10 μM). The number of animals ranges from 51 to 173 per cohort. See Table S12 for statistics.

The maximum effect size observed in our study was induced by *ZINC000019802386*, a compound identified by our machine learning model (+74%). This extension is comparable to the best‐performing compounds annotated in DrugAge (Alavez et al. 2011; Barardo et al. 2017; Miller and Roth 2007; Miller et al. 2022). *ZINC000019802386* is structurally similar to Octoclothepin (Tanimoto similarity, *T* = 0.45). There are no known activities against the targets in our GT clusters for *ZINC000019802386*, but close analogs interact with DRD1, DRD2, DRD3, and HTR2A. To experimentally verify the polypharmacological properties of the hits, we selected two structurally distinct compounds, ziprasidone and zotepine, which bind targets in at least two of the three identified clusters (Figure 2C). We tested their effect in four loss‐of‐function (*lof*) mutants or knockouts (*ko*) most closely related to the annotated mammalian GPCRs: two serotonin receptor mutants, *ser‐4*(*lof*) and *ser‐5*(*ko*), a dopamine receptor mutant *dop‐3*(*lof*), and an octopamine receptor mutant *ser‐3*(*lof*). Octopamine is the invertebrate equivalent of noradrenaline, and SER‐3 is analogous to mammalian adrenergic receptors (Alavez et al. 2011; Barardo et al. 2017; Miller et al. 2022; Perez‐Gomez et al. 2018; Petrascheck et al. 2007). Ziprasidone and zotepine were selected over the most active novel compound, *ZINC000019802386*, since the mammalian pharmacology of ziprasidone and zotepine has been established (Wishart et al. 2006).

As expected, the lifespan effects of both compounds depended on multiple GPCRs. Zotepine's lifespan extension was contingent upon *ser‐3*, *ser‐4*, and *ser‐5* (Figure 2D). Although there was still an extension, the effect sizes were less pronounced compared to the wild‐type N2, suggesting that binding to each receptor contributed partially and additively to the overall effect. However, the dopamine receptor *dop‐3* was not necessary for the longevity induced by zotepine. Similarly, ziprasidone's effect on extending the lifespan of *C. elegans* also required the same three GPCRs: *ser‐3*, *ser‐4*, and *ser‐5* (Figure 2E). Like zotepine, ziprasidone exhibited a reduced longevity effect in the *ser‐4*(*lof*) mutant. Interestingly, unlike zotepine, the lifespan extension by ziprasidone was eliminated in *ser‐3*(*lof*) and *ser‐5*(*ko*), indicating a critical dependency on these receptors.

A simple model in which GPCRs act as binary “on–off” switches, with ligands blocking signaling by the different receptors, only explains the interaction of zotepine with the receptor mutants. In this binary model, blocking signaling by each receptor modulates lifespan independently, partially contributing to the total effect size. This model does not explain the result for ziprasidone. More recent models suggest modulation of GPCRs by “ligand‐bias,” in which binding of the ligand causes the GPCR to recruit distinct downstream effectors such as G‐proteins, G‐proteins receptor kinases, or arrestins favoring one signaling modality over another (Schmid et al. 2008; Zhang et al. 2024).

Thus, a polypharmacological ligand may have different functional selectivity or ligand bias for each individual receptor (Berg and Clarke 2018). While the affinity of a ligand recruits it to a receptor, ligand bias modulates the downstream signaling, resulting in different outcomes for two ligands binding to the same receptor. Zotepine and ziprasidone require the same receptors for their full lifespan extension, but the receptors' quantitative contributions to the overall longevity effect differ. This observation suggests that the two compounds differ in their downstream effector response. Similar considerations also explain why GPCR ligands often do not phenocopy receptor knockout phenotypes and vice versa.

Until recently, polypharmacological action was accidental and often considered undesirable for two reasons. First, lead‐to‐drug optimization is far more complex when multiple targets are involved (Kabir and Muth 2022). Second, targets are often evaluated based on their genetics, mostly single gene knockouts or alleles, with far less data available on how different alleles interact. Consequently, polypharmacological design principles are challenging to implement in conventional drug discovery pipelines, in part because “off‐target effects” are often found detrimental. However, retrospective analyses of successful drugs show that their therapeutic efficacy or safety often depends on multiple targets, highlighting that unintended polypharmacology contributes to the success of some drugs (Bolognesi and Cavalli 2016; Lin et al. 2019). Our study establishes that computational strategies can solve the increased complexity of a polypharmacological approach and validate these complex predictions in vivo. The strengths of this approach in the design of geroprotectors were: (1) an exceptional hit rate of over ~70%, (2) an exceptional effect size in extending lifespan by over 40% across the entire set, and (3) the correct mechanistic prediction of a polypharmacological effect of the selected compounds.

The only shortcoming in the current study was that most novel structures did not extend lifespan. This observation could be explained in two ways. First, absorption, distribution, metabolism, excretion, and toxicity (ADMET) properties were not considered in our computational pipeline. Hence, the performance of our models may suffer when we deviate from well‐characterized structural motifs shared with approved drugs. Second, large neural networks, such as Chemprop used here, operate as “black boxes” that rely on limited experimental data typically available in biomedical datasets. We demonstrate that such systems function as “search engines” in chemical space and tend to favor compounds structurally similar to those active in the training dataset. These limitations may be overcome as more data become available for training models, network architectures improve, and more aggressive train/test set splits could be employed to achieve better generalization.

We focused our study on *C. elegans* , currently the only model organism with sufficient pharmacological lifespan data to train machine‐learning models for polypharmacological effects. All other model systems (mammalian, flies, and yeast) lack sufficient numbers of well‐established longevity targets for which the knockout of a target was shown to ablate the effect of a geroprotector. In addition, the short lifespan and small size of *C. elegans* make it possible to study complex phenomena such as polypharmacology, which would be exceedingly hard to do in mammals (Burdusel et al. 2024). However, given that aging in all organisms is a multifactorial decline of the biological network, the superior efficacy of polypharmacological agents is likely to be a general principle for aging‐related drug discovery and not restricted to *C. elegans* .

In summary, we combined the increasing arsenal of modern computational methods with the depth of available pharmacological data for longevity in *C. elegans*. We demonstrate that modern machine learning tools enable the development of polypharmacological geroprotectors to enhance efficacy in lifespan extension. Our study demonstrates how modern AI‐based tools may be used to transcend the traditional drug discovery paradigm by enabling the de novo design of polypharmacological geroprotectors. Moreover, our findings suggest that polypharmacological action is not just advantageous but a necessary step toward developing exceptionally efficacious geroprotectors.

## 1 Methods

### 1.1 Dataset Preparation

The *C. elegans* lifespan training data were taken from the results of screening of the LOPAC by Ye et al. (2014). The SMILES (Simplified Molecular Input Line Entry System) strings for compounds were retrieved from the PubChem database using the web interface (Kim et al. 2018). We derived hashed International Chemical Identifiers (InChIKeys) from the SMILE strings. For the compounds that existed in salt forms, we also included the InChIKeys for the parent compounds. Using these InChIKeys, we queried ChEMBL web services (Davies et al. 2015) to retrieve compound activity data. If no activity records matched a full InChIKey, we performed a relaxed search using only the first 14 characters of the InChIKey. Compounds with low activities were filtered out using the pChEMBL metric (Bento et al. 2014), representing the negative logarithm of activity values (e.g., IC_50_, EC_50_) in molar units. We applied the threshold of pChEMBL ≥ 5, resulting in 1029 of 1275 LOPAC compounds meeting this criterion. The activity data were further filtered out by selecting binding (B) and functional (F) assays with outcomes related to inhibition (Ki, IC_50_, Kd, etc.). Protein targets were clustered based on protein sequence similarity using the software suite MMseqs2 (Steinegger and Soding 2017) with a sequence identity threshold of 0.5. Within each cluster of similar targets, activity values were aggregated using the minimum pChEMBL value for a cluster member. These were subsequently binarized at the threshold of pChEMBL ≥ 7. A list of clusters and their members is shown in Table S4. This process yielded 1256 LOPAC compounds with known chemical structures and 281 targets, among which 594 compounds demonstrated activity on at least one target (Table S5). Compounds from LOPAC screening with significant positive effects on lifespan were denoted as LS+.

The GPCR dataset for training a neural network was also retrieved from the ChEMBL web services. We extracted all inhibition assays for the selected GPCR targets (listed in Table S7). Data were binarized using the threshold of 7 for the pChEMBL value.

### 1.2 Targets Discovery From the LOPAC Library Screening

Based on binarized activity data, we developed a series of predictive models for identifying compounds with lifespan‐extending effects (LS+ label). Standard classification metrics—precision, recall, and the Matthews correlation coefficient (MCC)—were used for model evaluation (Table S1). MCC was particularly useful for imbalanced datasets, as it evaluates both positive and negative predictions comprehensively (Chicco and Jurman 2020).

The first class of predictors is based on the hypothesis that lifespan extension is associated with binding to one single cluster. We predicted a compound to have a positive LS+ label if it had a non‐zero activity label for a selected cluster.

The second class of predictors utilized the hypothesis that a compound extends lifespan if it binds to multiple clusters, regardless of the cluster. Here, we summed the activity labels for each ligand and predicted a positive LS+ if the sum exceeded a threshold N (N varied from 2 to 20; Table S2).

The third class of predictors combined these two hypotheses and predicted a compound to have a positive LS+ label if it binds to multiple but specific clusters. To find the combinations of these clusters, we generated combinations *C*(*G*, *M*), where *G* is the number of all clusters equal to 281, and *M* is the number of selected clusters ranging from 2 to 5. Here, the compound is predicted as LS+ if it is simultaneously active for all members in the combination. As we assumed that activity data in the Chembl was incomplete, we also tried to weaken the binding requirement to all clusters when combining several clusters. The weakened binding requirement was expressed as a logic rule “*m* in *M*,” where *m* is the number of simultaneously active targets, and *M* is the size of a tuple. For example, the best predictor in this work had a logic rule “2 in 3,” indicating that a compound must be active on at least two out of three specific clusters (drd2, hrh1, htr6) to be classified as LS+. See the list of the best predictors in the Table S3.

### 1.3 Graph Neural Network for Predicting Molecular Interactions

We employed Chemprop (Heid et al. 2024), a GNN, to predict compound binding affinities to targets using data from ChEMBL. Chemprop utilizes a Directed Message Passing Neural Network, where molecular graphs are constructed from SMILES strings: atoms serve as nodes, and bonds as edges. The model generates atomic embeddings by processing node and edge features of neighboring atoms, which are then aggregated into a single molecular embedding. Chemprop optionally supports including pre‐computed molecular features to train molecular embeddings for better performance. The pre‐computed feature set (listed in Table S6) was adapted from the original Chemprop implementation by removing highly correlated descriptors to improve computational efficiency. The features were calculated from SMILES strings with the RDKit open‐source package (Greg Landrum et al. 2024) We processed the concatenated trained and pre‐computed molecular embeddings through a feed‐forward neural network (FNN) with DenseNet‐like skip connections (Haung et al. 2017), enabling each layer to access features learned by all preceding layers. The output of the FNN was a vector of probabilities of whether a molecule binds to one of the GPCR targets listed in Table S7. We employed cross‐entropy loss to handle the multi‐task classification problem, and missing binding data were excluded during loss computation.

We performed a train‐validation‐test split using the Butina clustering algorithm (Butina 1999) with a Tanimoto similarity cutoff of 0.8 and a ratio of 0.8/0.1/0.1. Hyperparameter optimization was performed using a grid search based on the area under the receiver operating characteristic curve (ROC‐AUC) from prediction scores on the test set. The final architecture and performance metrics are detailed in Tables S8 and S9.

### 1.4 Compound Selection for Experimental Validation

We applied the trained Chemprop model to identify candidate compounds to predict binding activities in the ZINC20 in‐stock subset (Irwin et al. 2020) against the selected GPCR targets. We selected compounds predicted to exhibit polypharmacological effects, specifically those that bound at least two of the following three clusters: drd2, hrh1, and htr6. We filtered candidates based on Quantitative Estimate of Druglikeness (QED) scores (Bickerton et al. 2012) and obtained 66 candidates for the experiment (Table S10). We applied the Butina clustering algorithm to ensure structural diversity and selected 16 unique, potentially active compounds for experimental validation.

### 1.5 *C. elegans* Strains

The Bristol strain (N2) was used as the wild‐type strain. The following strains used in this study were obtained from the Caenorhabditis Genetic Center (CGC; Minneapolis, MN): RB1631 [s*er‐3*(*ok2007*)] [referred to a *ser‐3*(*lof*) in the text], AQ866 [*ser‐4*(*ok512*)] (referred to a *ser‐4*(*lof*) in the text), and BZ973 [*dop‐3*(*ok295*)] [referred to a *dop‐3*(*lof*) in the text]. The strain VV212 [*ser‐5*(*vq1*)] [referred to as *ser‐5*(*ko*) in the text] was generated by Perez‐Gomez et al. (2018).

### 1.6 Lifespan Assays

Lifespan assays were conducted in 96 well plates in a liquid culture, as outlined in Clay and Petrascheck (2020). Each permutation (strain–drug) was tested in trials of 50–100 animals in each trial. These numbers have sufficient power to detect differences in lifespan of 20% or more in more than 95% of the time (Petrascheck and Miller 2017). Animals were treated with drugs on day 1 of adulthood to avoid any developmental effects. Lifespan experiments were conducted blind. Statistical analysis was performed using the Mantel–Haenszel version of the log‐rank test, and data are summarized in Tables S11 and S12.

## Author Contributions

K.A., K.A.D., and O.B. conducted the computational analysis of the LOPAC structures, developed and tested the computational strategies to predict polypharmacological geroprotectors. K.J.C. and M.P. conducted the *C. elegans* lifespan experiments and conducted the statistical analysis of the lifespan data. P.O.F. and M.P. directed the project. P.O.F., M.P., K.A., and K.J.C. wrote the paper.

## Conflicts of Interest

K.A., K.A.D., O.B., and P.O.F. are employed by Gero and declare no conflicts of interest. K.J.C. and M.P. declare no conflicts of interest.

## Supporting information


**Table S1.** Top 10 predictions of LS+ by an activity on a single cluster (MCC—Matthews correlation coefficient).
**Table S2.** Prediction of LS+ by the number of activity records per ligand.
**Table S3.** Prediction of LS+ by an activity on a combination of clusters.
**Table S4.** Clustering of ChEMBL protein targets by similarity.
**Table S5.** Compound with known activities against the selected clusters.
**Table S6.** Molecular features and RDKit implementation used in Chemprop training.
**Table S7.** Molecular targets with gene symbols used as Chemprop classification tasks.
**Table S8.** Model hyperparameters and training parameters.
**Table S9.** Classification metrics on the test data subset.
**Table S10.** List of selected compounds in the virtual screening of the ZINC library.
**Table S11.** Summary of lifespan screening statistics.
**Table S12.** Summary of lifespan mutant statistics.

## Data Availability

All data that support the findings of this study are available in the provided Excel, and details on the computational pipeline will be made available at: gero‐ai/polypharmacological_longevity·GitHub.
